# CD335 (NKp46)^+^ T-Cell Recruitment to the Bovine Upper Respiratory Tract during a Primary Bovine Herpesvirus-1 Infection

**DOI:** 10.3389/fimmu.2017.01393

**Published:** 2017-10-23

**Authors:** Rahwa A. Osman, Philip John Griebel

**Affiliations:** ^1^Vaccinology and Immunotherapeutics Program, School of Public Health, University of Saskatchewan, Saskatoon, SK, Canada; ^2^Vaccine and Infectious Disease Organization-International Vaccine Centre (VIDO-Intervac), University of Saskatchewan, Saskatoon, SK, Canada

**Keywords:** bovine herpesvirus-1, chemokines, interferon-γ, lymphocyte recruitment, non-conventional T-cells

## Abstract

Bovine natural killer (NK) cells were originally defined by the NK activation receptor CD335 [natural killer cell p46-related protein (NKp46)], but following the discovery of NKp46 expression on human T-cells, the definition of conventional bovine NK cells was modified to CD335^+^CD3^−^ cells. Recently, a bovine T-cell population co-expressing CD335 was identified and these non-conventional T-cells were shown to produce interferon (IFN)-γ and share functional properties with both conventional NK cells and T-cells. It is not known, however, if CD335^+^ bovine T-cells are recruited to mucosal surfaces and what chemokines play a role in recruiting this unique T-cell subpopulation. In this study, bovine herpesvirus-1 (BHV-1), which is closely related to herpes simplex virus-1, was used to investigate bovine lymphocyte cell populations recruited to the upper respiratory tract following a primary respiratory infection. Immunohistochemical staining with individual monoclonal antibodies revealed significant (*P* < 0.05) recruitment of CD335^+^, CD3^+^, and CD8^+^ lymphocyte populations to the nasal turbinates on day 5 following primary BHV-1 infection. Dual-color immunofluorescence revealed that cells recruited to nasal turbinates were primarily T-cells that co-expressed both CD335 and CD8. This non-conventional T-cell population represented 77.5% of CD355^+^ cells and 89.5% of CD8^+^ cells recruited to nasal turbinates on day 5 post-BHV-1 infection. However, due to diffuse IFN-γ staining of nasal turbinate tissue, it was not possible to directly link increased IFN-γ production following BHV-1 infection with the recruitment of non-conventional T-cells. Transcriptional analysis revealed CCL4, CCL5, and CXCL9 gene expression was significantly (*P* < 0.05) upregulated in nasal turbinate tissue following BHV-1 infection. Therefore, no single chemokine was associated with recruitment of non-conventional T-cells. In conclusion, the specific recruitment of CD335^+^ and CD8^+^ non-conventional T-cells to viral-infected tissue suggests that these cells may play an important role in either the clearance of a primary BHV-1 infection or regulating host responses during viral infection. The early recruitment of non-conventional T-cells following a primary viral infection may enable the host to recognize viral-infected cells through NKp46 while retaining the possibility of establishing T-cell immune memory.

## Introduction

Bovine herpesvirus-1 (BHV-1), a member of *Alphaherpesvirinae* subfamily, is an important pathogen contributing to bovine respiratory disease complex in young calves. BHV-1 causes a rhinotracheitis in the upper respiratory tract (URT) (1) and, similar to other alphaherpesviruses in humans, pigs and horses, causes a lytic infection of mucosal epithelial cells (2) followed by a latent infection in the peripheral nervous system. The primary sites of BHV-1 infection in the URT include the nasal turbinates, pharyngeal tonsils, and trachea (3). Previous studies suggested that interferon (IFN) does not play a major role in the clearance of a primary BHV-1 infection (4) but cytotoxic cell-mediated immune responses mediated by macrophages, neutrophils, natural killer (NK) cells, and cytotoxic T-lymphocytes (CTLs) may contribute to viral clearance (5–7).

Natural killer cells are non-antigen-specific innate lymphocytes that respond rapidly to both infectious and non-infectious challenges. NK cells express both activation and inhibitory receptors. These heterologous receptors include killer-cell immunoglobulin-like receptors and natural cytotoxicity receptors (NCRs) such as natural killer cell p46-related protein (NKp46) [natural cytotoxicity triggering receptor 1 (NCR1) or CD335], NKp30, and NKp44 (8). CD335 is the only NK receptor currently characterized for bovine NK cells (9) and is an activating receptor on NK cells, which binds ligands and initiates signaling that activates cytotoxic responses. This was demonstrated by activation of human NK cell cytotoxicity following NKp46-binding of hemaglutinin of influenza viruses (10). This activation signal results in the release of cytotoxic granules, which kill target cells through the combined action of perforin and granzyme (11).

CD335 was originally described as a bovine NK cell-specific receptor (9) but a small subpopulation of bovine T-cells have also been identified that co-express CD335 (9, 12–14). CD335^+^CD3^−^ cells are now defined as classical or conventional NK cells and lymphocytes that co-express CD3 and CD335^+^ are described as non-conventional T-cells. Multiple non-conventional T-cells have been reported in several mammalian species, including humans (15), mice (16), pigs (13), and bovine (12). The discovery of reprogrammed human CTLs that co-express CD3 and NKp46 in celiac disease (15) highlighted the existence of T-cells acquiring NCRs previously associated with NK cells. Other non-conventional T-cells include natural killer T (NKT) cells and mucosal-associated invariant T (MAIT) cells that co-express CD3 and NCRs.

Natural killer T-cells were first discovered in mice (17) and characterized as a T-cell subpopulation expressing NK1.1 and αβT-cell receptors (17, 18). In species that do not express NK1.1, the term NKT has been used to refer to T-cells which co-express NK cell receptors (19). NKT cells studied in humans and mice were shown to express an invariant T-cell receptor (TCR) and were termed invariant (i)NKT cells. This population recognized a limited repertoire of ligands relative to the extensive repertoire of conventional MHC-restricted T-cells. NKT cells also recognize lipid ligands complexed with the non-MHC surface molecule, CD1d, and were also referred to as “CD1d-restricted” T-cells (20). CD1d is absent in cattle (21) but bovine T-cells co-expressing CD335 do recognize lipid ligands by a CD1d-independent mechanism (22).

Mucosal-associated invariant T-cells were first observed in human blood by Porcelli et al. (23) as unconventional αβT-cells with invariant TCRα chain and semi-invariant TCR repertoire. These non-conventional T-cells have since been identified in mice and found to be enriched at mucosal surfaces (24). MAIT cells recognize antigens in the context of a non-classical-MHC molecule, MR1 (24). Recent studies have shown that MAIT cells have antimicrobial functions (25) and recognize vitamin B metabolite ligands (26). MAIT cells have not been characterized in cattle but bovine non-conventional T-cells that co-express CD335 have been shown to have a cytotoxic effector function with parasite-infected cells and secrete IFN-γ (12). No information is available, however, regarding the role of these cells in controlling infections at mucosal surfaces.

Homing of innate and adaptive lymphocytes to sites of viral infection is crucial for effective cell-mediated immune responses and clearance of viral-infected cells. Different non-conventional T-cell subsets home to specific tissues based on their expression of chemokine receptors and intrinsic tissue responses to pathogens or other danger signals (27). Murine iNKT cells express CCR7, CXCR3, CXCR6, CCR4, and CCR6 chemokine receptors (28), of which CCR4 (29) is important for pulmonary localization. CXCR6, CCR1, and CCR6 are expressed by human NKT cells (30) but the chemokine receptors expressed by bovine non-conventional T-cells and the chemokines involved in their recruitment to specific tissues has yet to be determined. Previous studies demonstrated that non-MHC-restricted lymphocytes were recruited to the lungs of calves following BHV-1 infection (7) but it was not determined whether these cells were conventional NK cells or non-conventional T-cells. It is not known if non-conventional T-cells may be recruited to bovine mucosal surfaces since previous studies of bovine non-conventional T-cells were limited to cells isolated from blood (12, 21, 22).

In this study, we demonstrate that non-conventional T-cells co-expressing CD335 and CD8 are recruited to the bovine URT following an experimental BHV-1 infection and these non-conventional T-cells are the major effector cell population recruited within 5 days post-infection (pi). Our analysis of chemokine gene expression at the site of viral infection also identified multiple chemokines that may be involved in the highly specific recruitment of this non-conventional T-cell population. Thus, CD335^+^ and CD8^+^ bovine non-conventional T-cells appear to be the primary effector cell population involved in the early immune response to a BHV-1 URT infection.

## Materials and Methods

### Animals

Female and castrated male, 5- to 6-month-old, crossbred (Angus X Hereford) calves (*n* = 30) were purchased from a single commercial herd. Calves were identified as seronegative for BHV-1 by screening with a recombinant, truncated glycoprotein D antibody capture ELISA (31). Calves were weaned, transported to the research facility, and adapted for 2 weeks to a diet of free choice hay and 0.5 kg oats/day. During the adaptation period, calves were housed as a single group and there was no contact with other cattle. The average weight of calves was 232 kg (range = 174 and 242 kg), and the animals were housed in a single pen throughout the study. All experimental procedures were conducted according to the Guide to the Care and Use of Experimental Animals, provided by the Canadian Council on Animal Care and the experimental protocols were approved by the University of Saskatchewan Animal Care Committee (Protocol #19940211 and 19940218). The animals used for tissue collection were the same animals used to analyze virus shedding, IFN gene expression, and the expression of IFN-stimulated genes in a previous study (4). In our previous study, we demonstrated that all infected animals shed greater than 10^5^ infectious virus particles/ml of nasal secretion at the time tissues were collected between days 3 and 7 pi (4). BHV-1 infection of nasal turbinate mucosal epithelium was also confirmed by immunohistochemical (IHC) staining (4).

### Experimental Infection and Sample Collection

Six (6) of the 30 calves served as uninfected controls and tissue samples were collected from the control animals immediately prior to challenging the remaining 24 calves with BHV-1. This ensured there was no potential exposure of control calves to BHV-1. The 24 infected calves were aerosol challenged with BHV-1 isolate 108 (5 × 10^7^ pfu/animal) on experimental day 0 as previously described (32). A clinical veterinarian, blinded to treatment group, examined calves daily and recorded body weight and temperature. For tissue collection, cohorts of six calves were randomly selected and euthanized with an intravenous injection of Euthanyl (240 mg/ml; Bimeda-MTC, Canada) on days 3, 5 7, and 10 pi. Tissue samples were collected within 15–20 min after euthanasia, and three replicate samples were immediately placed in RNAlater or 10% buffered formalin. Tissue samples from nasal turbinates were collected 10–12 cm from the external nares of all calves (Figure S1 in Supplementary Material). A 4–5 mm^2^ piece of nasal turbinate was placed on the surface of a 4–5 mm^2^ and 1-mm thick slice of fresh liver tissue with the epithelial surface of the turbinate supported by the liver. This protected the mucosal epithelium from damage during cryosectioning. Tissue blocks were snap-frozen in liquid nitrogen and stored at −80°C until tissues were used for cryosectioning and IHC staining. Formalin-fixed tissues were used for histology and IHC detection of BHV-1 proteins. Tissues fixed with RNAlater were stored at −80°C until RNA was extracted for qRT-PCR analysis of bovine chemokine gene expression.

### Monoclonal Antibodies (mAbs)

The mAbs used for IHC and immunofluorescence (IF) and relevant isotype controls are listed in Table 1, which provides details on mAb specificity and the concentration of each mAb used for staining.

**Table 1:** Monoclonal antibodies used for Immunohistochemistry and immunofluorescence.

CD	Target molecule	Target population	Isotype	mAb clone	Conc.	Source
CD3	TCR complex	Pan-T-cells	IgG1	MM1A	667 ng/ml	VMRD
CD4	CD4	T_H_ cells, DC	IgG1	CACT138A	10 µg/ml	VMRD
CD8	CD8α	CTLs, DC	IgG1	CACT80C	5 µg/ml	VMRD
CD335	NCR1, NKp46	Innate lymphoid cells	IgG1	AKS1	10 µg/ml	Bio-Rad
sIgA	Surface IgA	IgA plasma cells	IgG1	BIG312D3	125 ng/ml	VMRD
Interferon (IFN)-α	IFN-α	IFN-α	IgG1	Ascites	1:400 dilution	VIDO
IFN-γ	IFN-γ	IFN-γ	IgG1	7B6	2 µg/ml	LSBio
Isotype control			IgG1	MG100	5 µg/ml	Life Tech.

*CD, cluster of differentiation; TCR, T-cell receptor; T_H_ cells, T-helper cells; DC, dendritic cells; CTLs, cytotoxic T-lymphocytes; NCR1, natural cytotoxicity triggering receptor 1; NKp46, natural killer cell p46-related protein*.

### Lymphocyte Phenotyping with Immunohistochemistry

Three 4–5 mm^2^ nasal turbinate tissue samples were collected from each animal and tissue blocks were randomly selected for cryosectioning. Tissue blocks were sectioned at −20°C, and 5-µm tissue sections were cut using a cryostat (DAMON/IEC Division, Microtome 3398) and mounted on Superfrost Plus slides (Fisherbrand#12550215, ON, Canada). Serial tissue sections were selected for IHC staining on the basis of optimum tissue morphology. Tissue sections were fixed in chilled 100% acetone for 8 min and air-dried for 20 min prior to storage at 4°C. Indirect-immunolabelling was performed as described previously (33) to visualize the location and distribution of IFN-α and IFN-γ in tissue sections and to perform morphometric analyses of the frequency of CD335^+^, CD3^+^, CD8^+^, and CD4^+^ cells in nasal turbinates before infection (day 0) and on day 3, 5, 7, and 10 post-BHV-1 infection. Biotin-conjugated goat-anti-mouse IgG (Vector Laboratories, Burlingame, CA, USA #BA-9200) and the Vectastain Elite Avidin-Biotin complex Kit (Vector Laboratories, Burlingame, CA, USA #PK4000) were used to visualize bound mAbs. BHV-1 infection occurs primarily within mucosal epithelial cells of the nasal turbinate (4). Thus, boundaries set for morphometric analysis of individual lymphocyte populations included the mucosal epithelium and the underlying lamina propria region. Five contiguous microscopic fields (total area = 0.196 mm^2^) were analyzed within each tissue section, and cells with visible staining were manually counted using an Olympus CX31 microscope (Olympus, Center Valley, PA, USA) with a 40× objective. The first field counted was located at the upper left corner of the cryosection and four contiguous fields were then counted. The top margin of each field was defined by the external surface of the mucosal epithelium and included the underlying lamina propria. An Olympus BX51 microscope and DP70 digital camera system (Olympus, Center Valley, PA, USA) were used to capture images of IHC-stained cryosections.

### Immunofluorescence

Dual-color staining of tissue sections was performed using fluorochrome-conjugated mAbs that reacted with bovine CD3, CD8, and CD335. The mAbs (Table 1) were conjugated with fluorochromes using Molecular Probes^®^ Antibody Labeling Kit (Thermofisher Scientific, Carlsbad, CA, USA), following the manufacturer’s instructions. The anti-bovine CD3 mAb was conjugated with Alexa Fluor^®^ 594. The anti-bovine CD8 mAb was conjugated with Alexa Fluor^®^ 488 and Alexa Fluor^®^ 594, so it could be used in appropriate combinations with the fluorochrome-conjugated CD3 and CD335 mAbs. IgG1 isotype control mAb (Table 1) was conjugated with Alexa Fluor^®^ 594 to be used for confirmation of staining specificity of the three other fluorochrome-conjugated antibodies. Direct fluorochrome labeling was necessary since the three mAbs are all IgG1 isotype (Table 1) and mAb reactivity after fluorochrome conjugation was confirmed by flow cytometric analysis of labeled blood mononuclear cells. Serial nasal turbinate cryosections were cut and fixed as described previously and stored at 4°C overnight. Acetone-fixed sections were brought to room temperature, air-dried for 30 min, and then rehydrated with phosphate buffered saline (PBS). The PBS solution was changed three times at 5 min intervals before slides were blocked by incubating with 10% normal goal serum for 3 h, followed by a 5 min wash with PBS. Tissue sections were then flooded with 200 µl of PBS containing 5% normal goat serum and one of the following combinations of fluorochrome-conjugated mAbs: CD3 and CD335; CD335 and CD8; or CD3 and CD8. Cryosections were incubated in the dark at room temperature for 2.5 h before washing twice for 5 min with PBS that was mixed with a magnetic stirrer. Tissue sections were counterstained by flooding with 200 µl 4′,6-diamidino-2-phenylindole (1 µg/ml methanol) (Life Technologies, Camarillo, CA, USA) for 10 min at room temperature to visualize cell nuclei and then washed for 5 min with PBS. Tissue sections were cover slipped with Cytoseal 60 mounting media (Thermofisher Scientific, Carlsbad, CA, USA) and stored overnight at 4°C before fluorescent imaging was completed. Confocal images were generated using a Leica TCS SP8 scanning confocal microscope (Leica Microsystems, Wetzlar, Germany), equipped with a 63× oil immersion objective and utilizing the 405 nm (UV) and 561 nm laser lines. Confocal images from a minimum of 15 microscopic fields were captured per sample, and a minimum of 100 CD335^+^, CD3^+^, and CD8^+^ cells were counted per tissue section. Tissue sections were analyzed for nasal turbinate samples collected from three animals on day 5 pi to determine the frequency of CD335^+^CD3^+^, CD335^+^CD8^+^, and CD3^+^CD8^+^ cells.

### RNA Extraction

RNAlater^®^ fixed nasal turbinate samples were homogenized as previously described (34) and total RNA was extracted using the RNeasy mini Kit (Qiagen Inc., Ontario, CA, USA) following the manufacturer’s protocol. RNA quality and total RNA were determined using the Agilent 2100 Bioanalyzer (Agilent Technologies, Santa Clara, CA, USA). Samples with an RNA integrity number of eight or higher were used to prepare cDNA.

### cDNA Synthesis

Total RNA (1 µg) was reverse-transcribed using qScript™ cDNA SuperMix (Quanta Biosciences™ Beverly, MA, USA), following the manufacturer’s protocol. cDNA samples were diluted in DNAse/RNAse free water for use as template in qRT-PCR reactions.

### qRT-PCR Primer Design and Validation

Primer pairs for chemokine transcripts (Table 2) were designed using Clone Manager 9.0 program (Sci-Ed Software). Primer specificity for individual chemokine genes was confirmed through generation of a single peak in the melting curve and detecting a single amplicon following gel electrophoresis of PCR products. PCR products were also cloned in the PCR 2.1 vector using the TA cloning kit (Life Technologies, Camarillo, CA, USA). Cloned products were sequenced with a CEQ 200XL DNA Analysis system (Beckman Coulter) to confirm product identity and size. Primer amplification efficiency (Table 2) was determined using standard curves as described previously (35).

**Table 2:** Primers for amplification of bovine genes.

Gene		Primer sequence^a^	Size^b^	Efficiency^c^
*CCL2 (MCP-1)*	FWD RV	TCGCTGCAACATGAAGGTCT TATAGCAGCAGGCGACTTGG	119	2.02
*CCL3 (MIP-1*α*)*	FWD RV	CGGCAGCTTTCTCGCAAAAT CCTCTCAGGCATTCAGCTCC	166	1.98
*CCL4 (MIP-1*β*)*	FWD RV	AGCTCTGCGTGACTGTCCTG AGGGTCTGAGCCCATTGGTG	86	2.15
*CCL5 (RANTES)*	FWD RV	GCTCCATGGCAGCAGTTGTC AGGTTCAAGGCGTCCTCCAC	128	2.12
*CCL19 (MIP-3*β*)*	FWD RV	AGGTGCCAACGACGCTGAAG TGAGGAGCAGGTAGCGGTAG	92	2.17
*CCL20 (MIP-3*α*)*	FWD RV	GACTGCTGTCTCCGATATAC GATGTCACAGGCTTCATTGG	90	1.98
*CCL21*	FWD RV	TGGTCCTGAGCATCCTTGTC TGGCGGGAATCTTCTTTCGG	114	2.07
*CXCL8 (IL-8)*	FWD RV	CGCTGGACAGCAGAGCTCACA TGCCAAGAGAGCAACAGCCAGC	106	2.06
*CXCL9 (MIG)*	FWD RV	AGTGGGAGAAACAGGTCAAC AAGTGGGAGCTCATGTAGTC	129	2.13
*CXCL10 (IP-10)*	FWD RV	AAGGGAAAGGGTGGCTCATC GGCTGGGACTTAGCACATTG	114	1.98
*CXCL-11 (IP-9)*	FWD RV	AGCAGCAACAAGCATGAGTG CCGTCCGCCTTTGAACATAG	100	2.10
β*-Actin*	FWD RV	CTAGGCACCAGGGCGTAATG CCACACGGAGCTCGTTGG	116	2.00
*GAPDH*	FWD RV	TGGAAAGGCCATCACCATCT CCCACTTGATGTTGGCAG	60	2.17
*RPS9*	FWD RV	CCTCGACCAAGAGCTGAAG CCTCCAGACCTCACGTTTGTTC	62	2.07

*^a^Sequence of forward (FWD) and reverse (RV) primers*.

*^b^Size of amplified PCR product*.

*^c^Efficiency of product amplification*.

### qRT-PCR Analysis of Chemokine Gene Expression

Gene expression data for individual samples was normalized relative to β*-actin*, which was confirmed to be stable over multiple time points and not affected by BHV-1 infection in nasal turbinates (data not shown). We also analyzed the expression of GAPDH and RPS9 reference genes. The expression of the three reference genes did not differ significantly when comparing samples collected at different time points following BHV-1 infection (data not shown) but β*-actin* transcription displayed the least variability among animals at each time point. The β-actin gene also had the lowest quantification cycle (Cq) value when comparing among the three reference genes and was therefore selected as the best reference gene for detection of genes with high transcriptional levels. qRT-PCR was performed using the PerfeCTa^®^ SYBR^®^ Green FastMix for iQ (Quanta BioScience, Beverly, MA, USA). Briefly, 9 µl of Perfecta SYBR green master mix (2×), 3 µl of the primer pair at 3.3 µM, and 3 µl cDNA template were added to a 15 µl final volume. The reaction was performed in BioRad iCycler iQ PCR detection system using the following program: 1 cycle at 95°C for 30 s, 45 cycles of 95°C for 15 s, 60°C for 30 s, and 72°C for 30 s. After cycling, the temperature was increased starting from 56°C at a rate of 1°C every 10 s to build a melting curve (40 times). Amplification data obtained for individual genes was expressed as Cq and was subtracted from the Cq of the β-actin reference gene to obtain ΔCq (ΔCq = Cq gene − Cq β-actin).

### Statistical Analysis

All statistical analyses in were performed using GraphPad software (Version 7.0a, La Jolla, CA, USA). A one-way ANOVA with Holm–Sidak’s multiple comparisons test was used to compare the frequency of leukocyte subpopulations in nasal turbinates before BHV-1 infection (day 0) with each day sampled pi. A Mann–Whitney test was used to analyze changes in the expression level of individual chemokine genes in nasal turbinates following BHV-1 infection when compared to preinfection (day 0) levels.

## Results

### IFN Production in Nasal Turbinate Tissue

**Figure 1:**
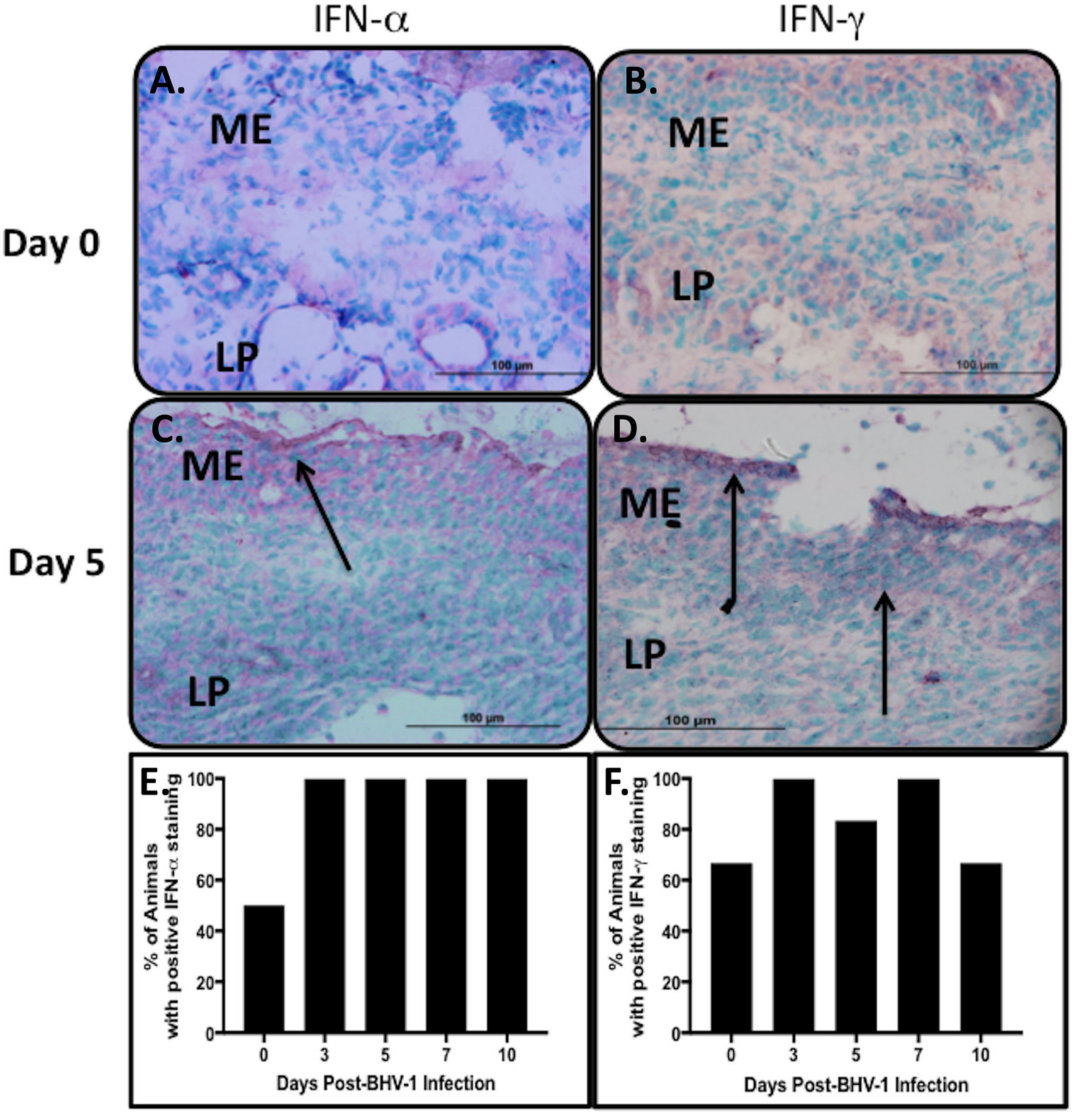
Type 1 and 2 interferon (IFN) secretion in nasal turbinates following a primary respiratory bovine herpesvirus-1 (BHV-1) infection of naïve calves. Immunohistochemical staining of cryopreserved nasal turbinate tissues with mouse anti-bovine IFN-α and anti-bovine IFN-γ monoclonal antibodies was used to visualize the location of IFN-α **(A,C)** and IFN-γ **(B,D)** protein. Representative tissue sections are presented to demonstrate an absence of staining in the uninfected control (day 0) and the pattern of staining 5 days (day 5) after BHV-1 infection. Diffuse staining of nasal turbinate epithelial surfaces (arrows) was observed in both uninfected and BHV-1 infected calves and the percent animals (*n* = 6/time point) with positive IFN-α **(E)** and IFN-γ **(F)** staining in nasal turbinates is shown. One tissue section was stained per animal. Brown color shows positive staining.

Previous studies confirmed IFN-α and -γ gene expression in nasal turbinates and the concentration of IFN in nasal secretions increased following a primary BHV-1 infection (4, 32). IHC staining of nasal turbinate cryosections with mAbs specific for bovine IFN-α and -γ was performed to determine if the production of these cytokines could be localized to specific cells within the tissue. IHC staining revealed, however, a diffuse staining pattern with the most intense staining associated primarily with mucosal epithelial cells (Figures 1C,D). Diffuse staining was occasionally observed throughout the lamina propria but there was no consistent pattern and staining could not be localized to individual cells. Isotype-matched mAbs were used to confirm the specificity of the staining observed at the mucosal surface and in the lamina propria (data not shown). Due to the diffuse and inconsistent staining pattern observed for IFN in tissue sections, a qualitative analysis was used to determine the frequency of positive staining in nasal turbinates collected from animals before and after BHV-1 infection. Some of the nasal turbinate samples collected from uninfected animals were positive for IFN-α (3/6 animals; 50%) and IFN-γ (4/6 animals; 60%) (Figures 1A,B). IHC staining of day 5 tissue sections revealed more intense IFN-α staining of the epithelial cell layer in all animals but only 80% (5/6 animals) of the tissue samples were positively stained for IFN-γ. The staining reaction was darkest at the epithelial surface and generally less intense in the lamina propria (Figures 1C,D). Positive staining for type I IFN on day 0 (Figure 1E) may reflect environmental exposure to other pathogens or commensal microbes that maintain a basal level of innate immune activation in mucosal epithelium of the URT. IFN-α is a potent activator of NK cells, which may explain the concurrent expression of IFN-γ (Figure 1F) if these innate immune cells are present in the tissue. Therefore, we investigated the types of lymphocytes present in nasal turbinates before and after BHV-1 infection.

### Lymphocytes Present in Nasal Turbinates following BHV-1 Infection

We previously demonstrated that bovine IFN-γ had limited direct antiviral activity, despite high levels of IFN-γ production during a primary BHV-1 infection (4). Thus, the role IFN-γ may play in clearing a primary BHV-1 respiratory infection is not known. One hypothesis is that increased IFN-γ production may reflect increased recruitment of either NK cells or T-cells to the site of viral infection and subsequent activation of these cells by Type I IFNs. Nasal turbinates are a site of BHV-1 infection and replication, and therefore, to test this hypothesis, nasal turbinate tissue was collected from BHV-1 infected calves. IHC was used to analyze the phenotype and frequency of innate and adaptive lymphocytes present in the tissue.

**Figure 2:**
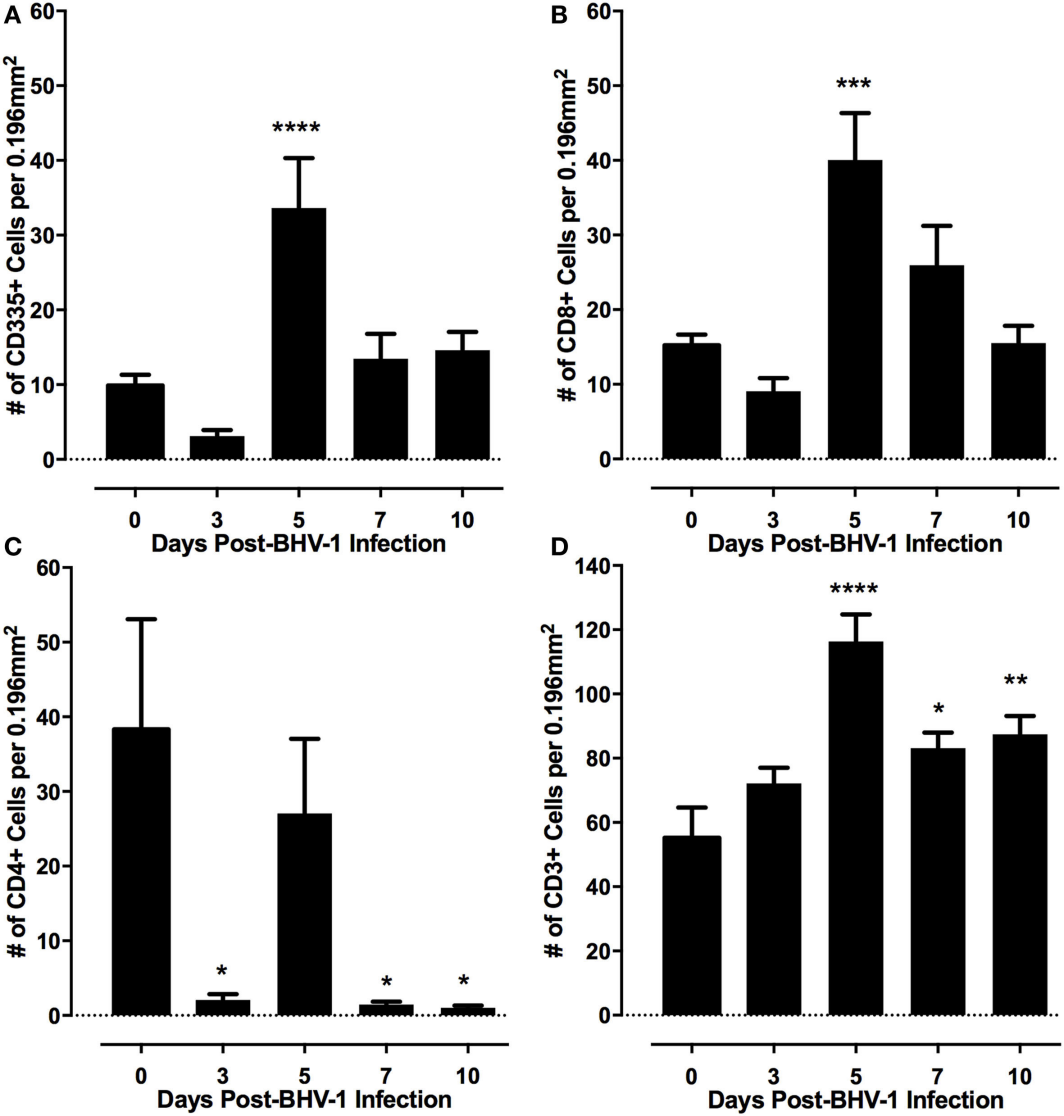
Frequency of lymphocyte subpopulations present in nasal turbinates following a primary bovine herpesvirus-1 (BHV-1) infection. Immunohistochemical staining was performed with monoclonal antibodies to identify CD335 **(A)** CD8 **(B)** CD4 **(C)**, and CD3 positive cells **(D)**. Morphometric analyses were used to quantify the frequency of each cell population located within the nasal turbinate intraepithelial and submucosal compartments. Data presented are the mean and SEM for values from tissue sections analyzed from six animals/time point following BHV-1 infection. One-way ANOVA was used to compare values relative to preinfection (day 0) levels and significant changes in frequency of leukocytes relative to day 0 are indicated as *P* < 0.05 (*), *P* ≤ 0.01 (**), *P* ≤ 0.001 (***), *P* ≤ 0.0001 (****).

**Figure 3:**
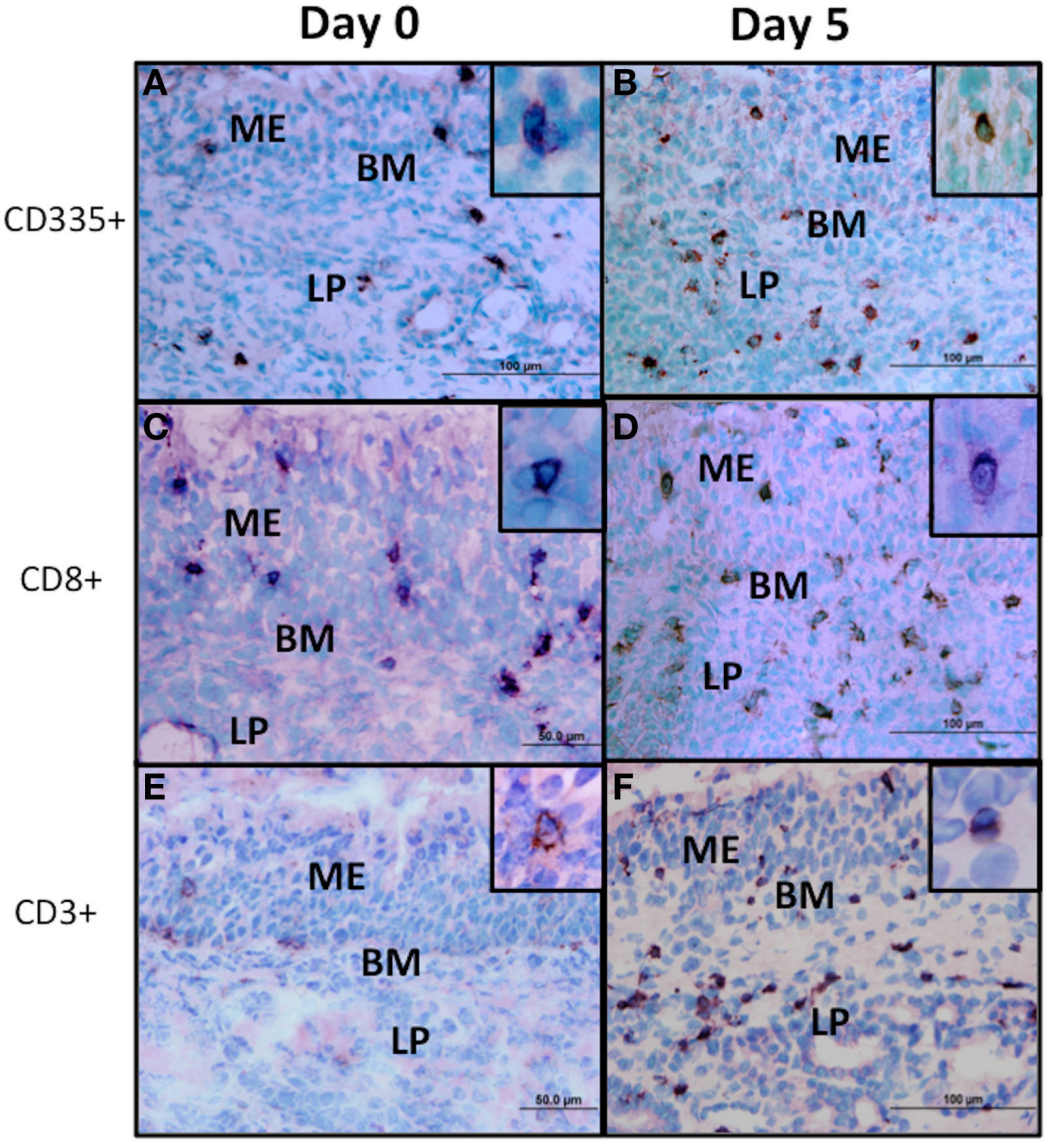
Immunohistochemical staining of lymphocyte subpopulations present in nasal turbinates following bovine herpesvirus-1 (BHV-1) infection. Sections from cryopreserved nasal turbinate were stained with monoclonal antibodies and an immunoperoxidase conjugate to visualize the distribution of CD335 **(A,B)**, CD8 **(C,D)**, and CD3 **(E,F)** positive cells before (day 0) and 5 days (day 5) after BHV-1 infection. No specific cellular staining was observed when using an isotype-matched irrelevant monoclonal antibody. Positive cells stain brown and the inset in each panel shows the detail of cellular staining. Panel images were captured at 60× magnification and the inset images were captured at 100× magnification. ME, mucosal epithelium; LP, lamina propria; BM, basal membrane.

Lymphoid populations present in nasal turbinates were first analyzed by staining for T-cells (CD3) and both conventional NK cells and non-conventional T-cells, which express CD335. Tissue sections were also stained for CD4 and CD8 which are markers for subsets of α/βTcR T-cells (36) and are also expressed on bovine NK cells and non-conventional T-cells (12). Morphometric analysis of tissue sections revealed that both T-cells (CD3^+^) and either conventional NK cells or non-conventional T-cells (CD335^+^) were present at low levels in nasal turbinates prior to BHV-1 infection (Figure 2). Cells staining for CD4 and CD8 were also present at low levels in the lamina propria prior to viral infection (Figures 2B,C and 3C; CD4 staining not shown). The number of CD335^+^, CD8^+^, and CD3^+^ cells present in the intraepithelial and lamina propria compartments increased significantly (*P* < 0.05) on day 5 pi (Figures 2C and 3) when compared to the number of cells present prior to infection (Figure 2). The increased number of cells present in nasal turbinates on day 5 then declined to preinfection levels on days 7 and 10 pi. In contrast, there was a significant (*P* < 0.05) decrease in the number of CD4^+^ cells on days 3, 7, and 10 pi (Figure 2). Cells staining for CD3 were the most abundant on day 5 pi, exceeding 100 cells per field (0.196 mm^2^) examined. The high frequency of CD3 cells on day 5 pi could not be accounted for by adding the total number of CD4^+^ and CD8^+^ cells, suggesting that other CD3 co-expressing cell populations may be recruited to the nasal turbinates following viral infection. γδTCR T-cells are a major T-cell subpopulation present at mucosal surfaces of ruminants (37) but the mAbs evaluated for bovine γδTCR staining reacted strongly with the mucosal epithelium. This non-specific staining precluded an accurate enumeration of all known T-cell subpopulations present in nasal turbinates following BHV-1 infection. IHC staining for IgA plasma cells also revealed consistently low numbers both before and after BHV-1 infection (data not shown).

### Non-Conventional T-Cells Are Recruited to Nasal Turbinates following BHV-1 Infection

**Figure 4:**
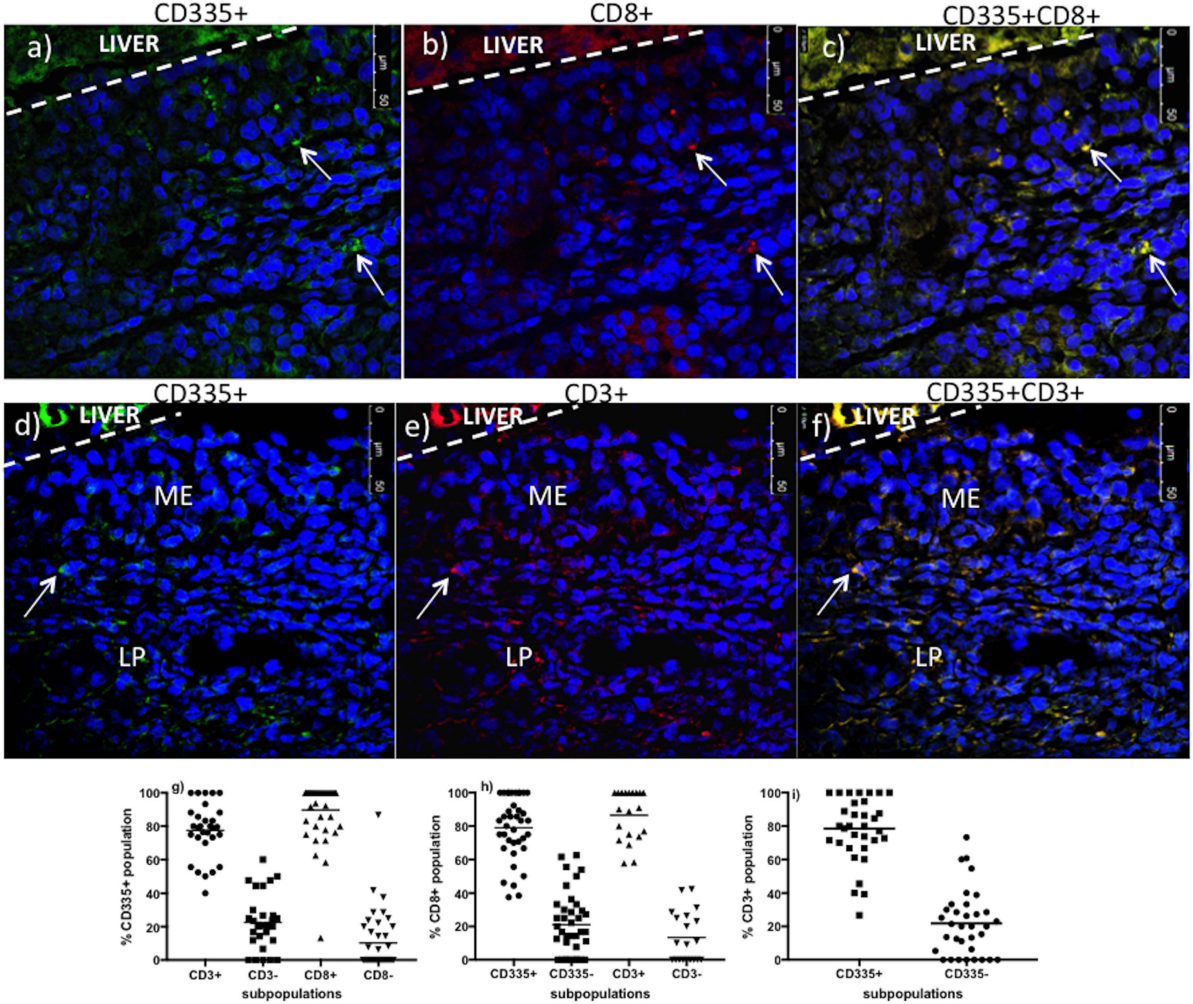
Co-expression of CD3 and CD8 on CD335^+^cells present in nasal turbinates on day 5 after a primary bovine herpesvirus-1 (BHV-1) infection. Nasal turbinate tissue was collected on day 5 post-infection and mounted on a piece of liver to maintain the morphology of the mucosal epithelium. Tissues samples were cryopreserved and tissue sections double stained by immunofluorescence. Staining for CD335 [**(A)**—stained green] and CD8 [**(B)**—red] was super-imposed to identify CD335^+^CD8^+^ cells [**(C)**—yellow]. Staining for CD335 [**(D)**—stained green] and CD3 [**(E)**—red] was super-imposed to identify CD335 cells co-expressing CD3 [**(F)**—yellow]. The liver portion of the cryosections is indicated (LIVER) and a white dashed line demarcates the separation between liver and nasal turbinate. 4′,6-diamidino-2-phenylindole was used as a counterstain to identify cell nuclei. Cell frequency data was generated through morphometric analyses. A minimum of 100 CD335^+^ **(G)**, CD8^+^ **(H)**, and CD3^+^ **(I)** cells were counted per animal and tissue samples from three animals were used to analyze the frequency of CD335^+^CD3^+^, CD335^+^CD8^+^, and CD3^+^CD8^+^ cells. Each dot represents the % cells of co-expressing the surface molecules indicated on the *y*-axis/per image/animal. ME, mucosal epithelium, LP, lamina propria. The images were captured at 63× magnification.

The CD335 mAb binds to the NKp46 receptor on innate immune cells and is involved in the activation of NK cells and target cell lysis (9).The NKp46 receptor is, however, expressed on a variety of lymphocytes, which includes both conventional NK cells (CD335^+^CD3^−^) and non-conventional T-cells (CD335^+^CD3^+^) that play key roles in host immune defenses and inflammation (12). Therefore, we further investigated the phenotype of the CD335 cells recruited to nasal turbinates during BHV-1 infection to determine if this might account for the large increase in CD3^+^ cells. A double staining protocol was optimized to determine if CD335^+^ cells co-expressed CD8 (Figures 4A–C) or CD3 (Figures 4D–F). On average, 77.5% of CD335^+^ cells present in nasal turbinates on day 5 pi co-expressed CD3 while 22.5% of the CD335^+^ cells expressed markers typical for conventional NK cells (CD335^+^CD3^−^) (Figure 4G). Further, 89.7% of CD335^+^ cells present on day 5 pi co-expressed CD8 (Figure 4G). Conversely, 78.5% of CD3^+^ cells present in the nasal turbinates on day 5 pi co-expressed CD335 while 21.8% of the CD3^+^ cells co-expressed the markers of conventional T-cells (CD3^+^CD335^−^) (Figure 4I). Furthermore, co-staining for CD8 and CD3 revealed 86.6% of CD8^+^cells recruited to the nasal turbinates on day 5 pi co-expressed CD3 (data not shown). Double staining for CD8 and CD3 also indicated that only 73.4% of CD3 cells co-expressed CD8, which verified that other CD3-expressing cell populations were recruited to the nasal turbinates following BHV-1 infection. The specificity of the fluorescent signal observed when dual-staining cells was confirmed by the absence of detectable fluorescent signal in the mucosal epithelium and lamina propria when tissue sections were staining with flourochrome-conjugated isotype control mAbs (Figure S2 in Supplementary Material). Thus, co-expression analysis revealed that the predominant population of lymphoid cells recruited to nasal turbinates on day 5 pi were characterized by co-expression of CD335 and CD3 and the majority of these non-conventional T-cells also co-expressed CD8.

### Chemokine Induction following BHV-1 Infection

**Figure 5:**
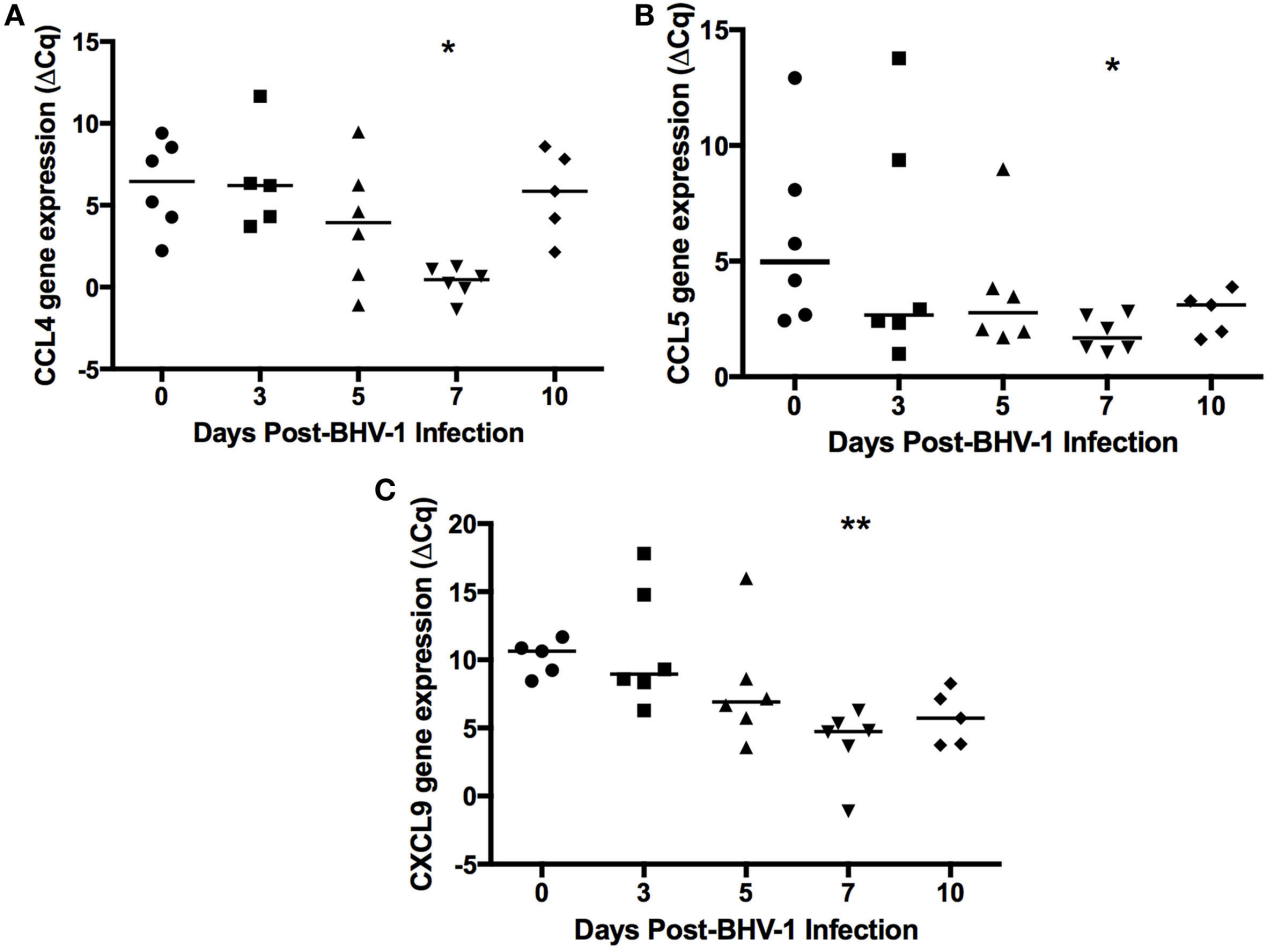
Expression of chemokines in nasal turbinate tissue following primary bovine herpesvirus-1 (BHV-1) infection. Nasal turbinate samples were collected (*n* = 6 animals/time point) prior to and following BHV-1 infection and RNA extracted for qRT-PCR analysis abundance of CCL4 **(A)**, CCL5 **(B)**, CXCL9 **(C)** gene transcript abundance. Gene expression was calculated as the change in threshold cycle (ΔCq) relative to β-actin and data presented are values for individual animals. The horizontal bar represents the mean value for animals sampled at each time sampled. A Mann–Whitney test was used to compare values relative to preinfection (day 0) levels. Significant changes in gene expression relative to day 0 are indicated: *P* < 0.0332 (*), *P* ≤ 0.0021 (**).

Human and mouse NKT cells express many different chemokine receptors that are involved in the recruitment and localization of NKT cells at sites of inflammation (20). Chemokine receptor expression has not been characterized for bovine NKT cells but the production of IFN-γ by human NKT has been shown to provide a positive feedback mechanism that enhances further NKT cell recruitment through induction of chemokines (38) Peak IFN-γ production following BHV-1 infection occurs on day 5 pi and this coincides with maximum recruitment of non-conventional T-cells (Figure 2). Therefore, we designed and validated qRT-PCR primers for known bovine chemokine genes (Table 2) to investigate whether BHV-1 infection altered the expression of chemokines that may be involved in recruitment of non-conventional T-cells. Among the 10 bovine chemokine genes analyzed, transcript levels for CCL4 (Figure 5A; *P* < 0.05), CCL5 (Figure 5B; *P* < 0.05), and CXCL9 (Figure 5C; *P* < 0.01) were significantly upregulated on day 7 pi. Thus, there did not appear to be a close temporal association between initial non-conventional T-cell recruitment to nasal turbinates and the onset of tissue expression of chemokine genes known to be involved in NKT cell recruitment.

## Discussion

CD335 (NKp46, NCR1) is commonly used to identify NK cells in cattle and other mammalian species such as sheep (14) pigs (13), humans (39), and mice (16). However, non-conventional T-cell subsets co-expressing CD335 have been reported in the blood, spleen, lungs, and other tissues of both healthy and infected animals (12, 13, 40). The current study provides the first demonstration that non-conventional T-cells co-expressing both CD335 and CD8 are the predominant lymphocyte population recruited to the URT following a primary BHV-1 infection. The frequency of CD335^+^ T-cells in bovine blood ranges between 0.1 and 1.7% of blood mononuclear cells and CD3^+^ cells usually comprise less than 10% of all CD335^+^ lymphocytes in blood (12). The frequency of CD335^+^ T-cells recruited to the URT following a primary BHV-1 infection was 77.5% of all CD335^+^ lymphocytes (Figure 4H) and 78.5% of all T-cells (Figure 4I). This observation supports the conclusion that there was a highly selective recruitment of these non-conventional T-cells. Recruitment of CD335^+^ T-cells to the lung of pigs was also reported following influenza infection (13), suggesting that CD335^+^ T-cells may play an important role in the early control of respiratory viral infections. Non-MHC-restricted cytotoxic cells were previously reported to be recruited to the lungs of calves following a primary BHV-1 infection but the phenotype of these cells was not determined (7). Thus, further phenotypic and functional analysis of immune cells recruited during primary viral infections may reveal that non-conventional T-cells provide an important early response to infection.

The majority of non-conventional T-cells recruited to the URT also co-expressed CD8 (Figure 4G) and bovine CD335^+^CD8^+^ T-cells were shown to produce IFN-γ when stimulated through CD3 (12). Recruitment of primarily CD8^+^ non-conventional T-cells to the URT is consistent with the previous observation that 75% of non-conventional T-cells in blood co-express CD8 (12). Maximum recruitment of non-conventional T-cells to the nasal turbinates was observed on day 5 post-BHV-1 infection which coincides with maximum IFN-γ secretion in nasal secretions (4). The IHC staining of nasal turbinates for IFN-γ revealed, however, a diffuse staining pattern that could not be localized to cells located in either the mucosal epithelium or submucosa (Figure 1D). Thus, it was not possible to directly link increased IFN-γ production following BHV-1 infection with the recruitment of a large number of non-conventional T-cells. Future studies will be necessary to develop methods to isolate this unique T-cell population from tissue following viral infection for the analysis of IFN-γ secretion or *in situ* analysis of IFN-γ transcript within individual cells may determine if T-cells co-expressing CD335 are a source of IFN-γ. It would also be interesting to determine if IFN-α is a potent activator of IFN-γ secretion by bovine non-conventional T-cells since peak production of both types of IFN occur on day 5 post-BHV-1 infection (4).

Innate and acquired immune responses to BHV-1 infection have been an area of research interest for over 40 years (41), and a variety of cell-mediated cytotoxic mechanisms have been implicated as important for the control and clearance of a primary BHV-1 infection (1, 7, 42). IFN-γ secretion has also been frequently used as an indicator of either NK cell or T-cell activation by BHV-1 proteins but these studies were frequently performed with lymphocytes isolated from blood (43). This is the first report that identifies effector lymphocyte populations recruited to mucosal surface of the URT following a primary BHV-1 infection. In this study, we combined IHC and IF analyses to demonstrate that CD8^+^ non-conventional T-cells were the primary lymphocyte population recruited early during a primary BHV-1 infection. Significant (*P* < 0.05) recruitment of this non-conventional T-cell population to the lamina propria was, however, limited to day 5 pi (Figure 2). A previous study in mice demonstrated that NK cell recruitment to lymph nodes following poxvirus infection was dependent on IFN-γ production (44). This observation may explain the coincidence of peak non-conventional T-cell recruitment to nasal turbinates with maximum levels of IFN-γ in nasal secretions (4). An alternative explanation may be that data showing recruitment of non-conventional T-cells was biased by sampling nasal turbinate tissues at a fixed site. BHV-1 infection in the URT of naïve calves occurs within discrete foci of mucosal epithelium (4). When non-conventional T-cells are first recruited from blood, they may be abundant throughout the lamina propria but the apparent decline in lymphocyte number throughout the lamina propria on days 7 and 10 pi (Figure 2) may reflect further recruitment and localization of non-conventional T-cells to foci of viral infection. IHC studies analyzing lymphocyte subpopulations recruited specifically to foci of BHV-1 infection in the URT may better define the kinetics of effector lymphocyte populations recruited during viral infection.

Chemotactic migration of non-conventional T-cell subsets has been examined in mice and humans but the chemokines involved in non-conventional T-cell recruitment in cattle is not known. Murine iNKT cells express CCR7, CXCR3, CXCR6, CCR4, and CCR6 chemokine receptors (28), of which CCR4 was critical for localization to the lung (29). CXCR6, CCR1, and CCR6 are expressed on the surface of human NKT cells (30). These studies indicate that non-conventional T-cells express multiple chemokine receptors and individual receptors may be involved in tissue-specific recruitment. Bovine CD335^+^CD2^+^ NK cells have been shown to express several chemokine receptors including CCR1, CCR8, CXCR6, and CX3CR1, while CD335^+^CD2^−^ NK cells expressed CCDR2, CCR5, CCR6, CCR7, CXCR3, CXCR4, and CXCR5 (45). Thus, bovine NK cells also have the capacity to respond to a broad range of chemokines. The expression of 11 known bovine chemokine genes was analyzed in nasal turbinate tissue collected following BHV-1 infection and CCL4, CCL5, CXCL9 were significantly (*P* < 0.05) upregulated (Figure 5). Transcript abundance for all three chemokine genes was greatest on day 7 pi and then remained at a similar level on day 10 pi. Significant chemokine gene expression on day 7 pi coincided with the highest level of BHV-1 replication and shedding in nasal secretions (4). If these chemokines are involved in non-conventional T-cell recruitment to viral infected mucosal epithelial cells then the temporal pattern of chemokine gene expression may explain the apparent decline in the abundance of these T-cell subsets in the lamina propria on days 7 and 10 pi. It is also interesting that maximum CXCL9 gene expression occurred on day 7 pi, which is 2 days after peak IFN-γ production in the URT (4). Increased expression of CXCL9, also known as monokine induced by IFN-γ (MIG; Table 2), is consistent with the strong IFN-γ response observed following BHV-1 infection (4). If CXCL9 is a chemokine involved in non-conventional T-cell recruitment then the IFN-γ response induced during BHV-1 infection may play an indirect role in recruitment of this specific effector cell population. Further clarification of which chemokines are involved in the specific recruitment of non-conventional T-cells will require an analysis of chemokine receptor expression on these cells.

## Conclusion

The present study demonstrates that CD335^+^CD8^+^ non-conventional T-cells are the predominant lymphocyte population recruited to the lamina propria of nasal turbinates within 5 days after a primary BHV-1 infection. Dual-color IF studies confirmed that over 75% of both CD335^+^ cells and CD3^+^ cells present in the lamina propria on day 5 pi could be classified as non-conventional T-cells. Although bovine CD335^+^CD8^+^ cells are a minor lymphoid population in blood (12), over 30 CD335^+^CD8^+^ T-cells/0.196 mm^2^ were present in bovine nasal turbinate tissue on day 5 pi. This represents a highly specific recruitment of non-conventional T-cells and suggests that CD335^+^CD8^+^ T-cells may be an important effector population for the control or clearance of a herpesvirus infection. The recruitment of these non-conventional T-cells was associated with increased expression of three different chemokines, indicating that this tissue-specific recruitment may involve multiple chemoattractant signals. Finally, the T-cell receptor repertoire of these cells needs to be determined before classifying these cells as either innate or adaptive T-cells.

## Ethics Statement

All experimental procedures were conducted according to the Guide to the Care and Use of Experimental Animals, provided by the Canadian Council on Animal Care and the experimental protocols were approved by the University of Saskatchewan Animal Care Committee (Protocol #19940211 and 19940218).

## Author Contributions

PG and RO were responsible for conception, design of the study, and writing the manuscript. RO designed and validated primers, performed experiments, and analyzed data. PG assisted in data interpretation. All authors read and approved the final manuscript.

## Conflict of Interest Statement

The authors declare that the research was conducted in the absence of any commercial or financial relationships that could be construed as a potential conflict of interest. The reviewer JH and handling editor declared their shared affiliation.
