# Supplementary material for: CD335 (NKp46)+ T-Cell Recruitment to the Bovine Upper Respiratory Tract during a Primary Bovine Herpesvirus-1 Infection
Source: Front Immunol. 2017 Oct 23;8:1393. doi: 10.3389/fimmu.2017.01393 (PMC5660870; doi:10.3389/fimmu.2017.01393)
Supplement: Supplementary file 1 [file Image_1.PDF]

Supplemental Figure 1

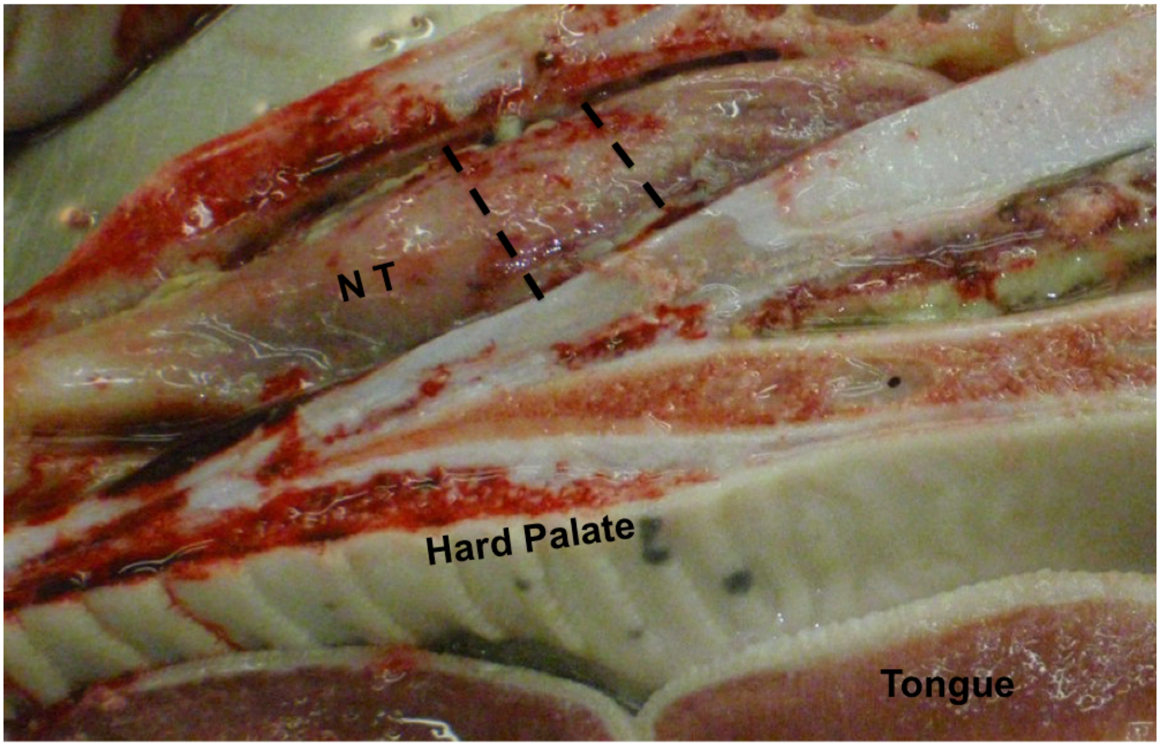

Supplemental Figure 1. Nasal turbinates in the upper respiratory tract of a 6 month old calf. A midsagittal cross-section of the head shows nasal turbinates located posterior to the external nares and photograph was taken on day 5 post-BHV-1 infection. Nasal turbinate samples were collected at the location indicated by the dashed lines on Day 0 (pre-infection) and Days 3, 5, 7 and 10 post-infection. Tissue samples were collected from 6 calves at each time point. N T = Nasal turbinates
