# Supplementary material for: CD335 (NKp46)+ T-Cell Recruitment to the Bovine Upper Respiratory Tract during a Primary Bovine Herpesvirus-1 Infection
Source: Front Immunol. 2017 Oct 23;8:1393. doi: 10.3389/fimmu.2017.01393 (PMC5660870; doi:10.3389/fimmu.2017.01393)
Supplement: Supplementary file 2 [file Image_2.PDF]

Supplemental Figure 2

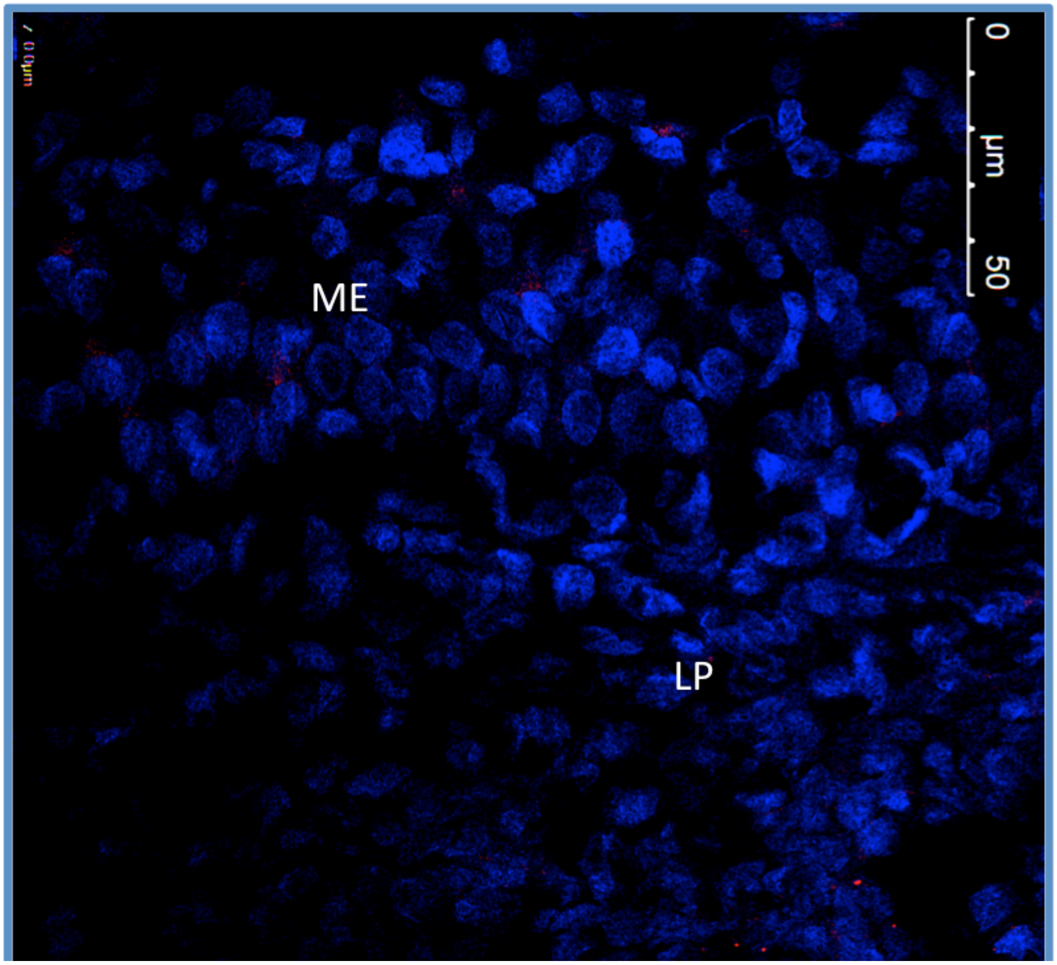

Supplemental Figure 2. IgG1 Isotype Control staining of nasal turbinate tissue section on day 5 post primary BHV-1 infection. Nasal turbinate tissue was collected on day 5 pi. Tissues sections were stained with an equivalent concentration of IgG1 isotype control directly conjugated to Alexa Fluor® 594 (Red fluorescence) and was used as a specificity control when dual-staining for CD335 versus CD3 and CD335 versus CD8 (Figure 4). DAPI was used as a counterstain to identify cell nuclei. ME = Mucosal Epithelium, LP = Lamina Propria. Images were captured at 63X magnification.
